# Early *versus* delayed initiation of renal replacement therapy for acute kidney injury: an updated systematic review, meta-analysis, meta-regression and trial sequential analysis of randomized controlled trials

**DOI:** 10.5935/0103-507X.20180054

**Published:** 2018

**Authors:** Fabio Tanzillo Moreira, Henrique Palomba, Renato Carneiro de Freitas Chaves, Catherine Bouman, Marcus Josephus Schultz, Ary Serpa Neto

**Affiliations:** 1 Departamento de Terapia Intensiva, Hospital Israelita Albert Einstein - São Paulo (SP), Brasil.; 2 Department of Intensive Care, Academic Medical Center; University of Amsterdam - Amsterdam, The Netherlands.; 3 Laboratory of Experimental Intensive Care and Anesthesiology, Academic Medical Center, University of Amsterdam - Amsterdam, The Netherlands.

**Keywords:** Acute kidney injury, Critically ill, Renal replacement therapy, Randomized controlled trial, Systematic review, Meta-analysis

## Abstract

**Objective:**

To evaluate whether early initiation of renal replacement therapy is associated with lower mortality in patients with acute kidney injury compared to delayed initiation.

**Methods:**

We performed a systematic review and meta-analysis of randomized controlled trials comparing early versus delayed initiation of renal replacement therapy in patients with acute kidney injury without the life-threatening acute kidney injury-related symptoms of fluid overload or metabolic disorders. Two investigators extracted the data from the selected studies. The Cochrane Risk of Bias Tool was used to assess the quality of the studies, and the Grading of Recommendations Assessment, Development and Evaluation (GRADE) approach was used to test the overall quality of the evidence.

**Results:**

Six randomized controlled trials (1,292 patients) were included. There was no statistically significant difference between early and delayed initiation of renal replacement therapy regarding the primary outcome (OR 0.82; 95%CI, 0.48 - 1.42; p = 0.488), but there was an increased risk of catheter-related bloodstream infection when renal replacement therapy was initiated early (OR 1.77; 95%CI, 1.01 - 3.11; p = 0.047). The quality of evidence generated by our meta-analysis for the primary outcome was considered low due to the risk of bias of the included studies and the heterogeneity among them.

**Conclusion:**

Early initiation of renal replacement therapy is not associated with improved survival. However, the quality of the current evidence is low, and the criteria used for -early- and -delayed- initiation of renal replacement therapy are too heterogeneous among studies.

## INTRODUCTION

Acute kidney injury (AKI) is a common condition in critically ill patients that results in fluid overload, acid-base disorders, electrolyte imbalances and azotemia. Despite several advances in the care of critically ill patients over recent decades, patients with AKI continue to have higher in-hospital mortality rates, especially when some form of renal replacement therapy (RRT) is needed.^(1-3)^

The optimal timing of RRT initiation remains unclear. The Kidney Disease: Improving Global Outcomes (KDIGO)-clinical practice guideline for AKI states that RRT should be initiated early when life-threatening conditions develop, such as fluid overload, hyperkalemia or acidosis.^(4)^ Otherwise, RRT initiation should be delayed and based on laboratory and clinical parameters, such as trends in laboratory tests, urine output, previous medical conditions and the patient's prognosis. While one systematic review and meta-analysis of previous randomized clinical trials showed that early RRT initiation is not associated with reduced mortality in patients with AKI,^(5)^ two recently published and well-powered randomized clinical trials (RCTs) showed conflicting results. A single-center RCT found early initiation to be superior to delayed initiation with respect to 90 day mortality.^(6)^ A multicenter RCT found no differences between early and delayed RRT initiation with respect to 60 day mortality.^(7)^ Interestingly, one cross-sectional survey performed in 24 intensive care units (ICUs) found that more than 90% of ICU physicians believe that early RRT initiation benefits every AKI patient, with a remarkable heterogeneity in what clinicians call 'early initiation'.^(8)^

We conducted an updated systematic review and meta-analysis of RCTs comparing early versus delayed RRT initiation. In addition to including the two new RCTs mentioned above,^(6,7)^ we conducted exploratory analyses using meta-regressions and a trial sequential analysis to assess the available power of the present analysis. We hypothesized that early RRT initiation is superior to delayed RRT initiation with respect to mortality.

## METHODS

### Search strategy

Research studies were identified via an electronic search of PubMed and Central (The Cochrane Library) through August 2016 by two investigators. The search strategy incorporated keywords and utilized the following Medical Subject Headings: (Acute kidney injury[MeSH] OR "acute renal"[ti] OR "acute kidney"[ti] OR kdigo[ti] OR critically ill[ti] OR intensive care unit[ti]) AND (Renal replacement therapy[MeSH] OR dialysis[ti] OR dialyzing[ti] OR dialyzed[ti] OR hemodialysis[ti] OR hemofiltration[ti] OR renal-replacement therapy[ti]) AND (Time to treatment[MeSH] OR Time factors[MeSH] OR Early[ti] OR earlier[ti] OR time[ti] OR timing[ti] OR accelerate[ti] OR accelerated[ti] OR accelerating[ti] OR acceleration[ti] OR late[ti]) AND (randomized OR clinical trial OR prospective). The title and abstract from all of the articles were scanned for relevancy. For potentially relevant articles, the full text was obtained for review. From these articles, as well as related reviews and meta-analyses, all references were inspected, and potentially relevant titles were hand searched. No further limitations were set on the query.

### Selection of research studies

The following three inclusion criteria were used: 1) RCTs of RRT; 2) adult patients with AKI; and 3) studies comparing early versus delayed initiation. Observational studies, retrospective studies, and RCTs studying the initiation of dialysis in patients with progressive chronic kidney disease or other indications rather than AKI were excluded.

### Data extraction and quality assessment of the studies

Two investigators extracted the data into a database developed for this particular dataset. If the investigators disagreed on data extraction, this was settled by discussion. The Cochrane Risk of Bias Tool was used to assess the studies' quality. Despite the description of the blinding of personnel, patients, or outcome assessors in our assessment of bias, we considered it for the classification of the studies for the following two reasons: because of the nature of the intervention, blinding investigators and healthcare personnel to the group allocation is not feasible; and blinding of the outcome assessors would not introduce a differential detection bias because the primary outcome assessed was mortality. We considered trials with lower risk of bias to indicate those at low risk of bias in all of the domains assessed.

### Definition of endpoints

The primary endpoint was mortality at longest follow-up, defined as all deaths during the admission period until the longest follow-up reported. Follow-up periods of mortality were highly variable and depended on the reported data in the retrieved articles. The secondary endpoints were as follows: in-hospital mortality; 28-day mortality; recovery of renal function at the longest follow-up (defined as dialysis independency at the longest follow-up reported); and complications potentially related to AKI or RRT, such as bleeding, catheter-related bloodstream infection, and thrombosis.

### Statistical analysis

For the meta-analysis, we considered all of the manuscripts included in the systematic review. All of the patients were analyzed in the study group to which they were randomized in the original study, i.e., the early or delayed RRT initiation arms (intention-to-treat principle). For dichotomous data, we calculated a pooled estimate of odds ratio (OR) with 95% confidence intervals (95%CI) in the individual studies using a random-effects model according to the DerSimonian-Laird method and graphically represented these results using forest plot graphs. The homogeneity assumption was measured by the *I^2^*, which describes the percentage of total variation across the studies due to heterogeneity rather than chance. *I^2^* was calculated from the basic results obtained from a typical meta-analysis as *I^2^* = 100% *x* (*Q* - df) / *Q*, where *Q* is the Cochran's heterogeneity statistic. A value of 0% indicates no observed heterogeneity, and larger values indicate increasing heterogeneity. For the primary outcome analysis, publication bias was addressed visually using a funnel plot, and the Grading of Recommendations Assessment, Development and Evaluation (GRADE) approach was used to test the overall quality of evidence.

Subgroup analyses were carried out by recalculating pooled OR estimates for the different subgroups as follows: the type of RRT (exclusively continuous *versus* intermittent or continuous) and the risk of bias (lower *versus* higher risk). These analyses were performed to test whether the overall results were affected by a change in the meta-analysis selection criteria. For the primary outcome and secondary outcome of renal function recovery at the longest follow-up, meta-regressions were performed using the year of publication, the percentage of patients receiving the continuous RRT method in the early arm and the time between randomization and RRT initiation in the early arm as covariates.

As the event size needed for a very precise meta-analysis is at least as large as that for a single optimally powered RCT, we calculated the optimal event size required for the primary endpoint in our meta-analysis considering a mortality rate of 55% in the delayed group, an expected treatment effect of 18%, 80% power, and a type I error of 5%.^(6)^ Thus, the observation of at least 1310 events would be needed. We performed a formal trial sequential analysis (TSA; TSA software version 0.9 Beta; Copenhagen Trial Unit, Copenhagen, Denmark) using the optimal event size to help construct the sequential monitoring boundaries for our meta-analysis, analogous to interim monitoring in an RCT.^(9)^ We established boundaries limiting the global type I error to 5%. As a sensitivity assessment, we also conducted TSA considering a stricter type I error of 1%. This more conservative approach may be appropriate for a meta-analysis of small trials.^(10)^

All analyses were conducted with Review Manager version 5.1.1, Statistical Package for Social Science version 20 (IBM SPSS Statistics for Windows, Version 20.0. Armonk, NY: IBM Corporation) or R version 2.12.0 (R Foundation for Statistical Computing, Vienna, Austria). For all analyses, two-sided p values < 0.05 were considered significant.

## RESULTS

**Figure 1:**
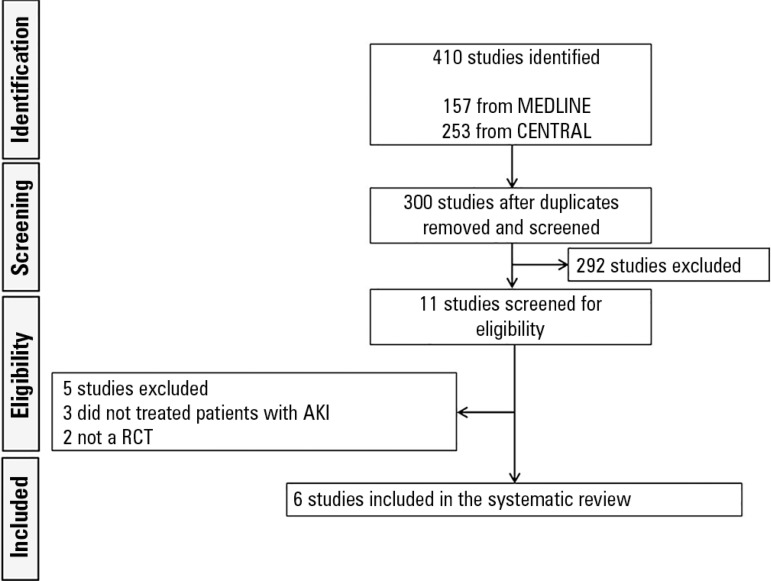
Flowchart of the study's search and selection process. AKI - acute kidney injury; RCT - randomized controlled trial.

The initial search yielded 410 articles: 157 from MEDLINE and 253 from CENTRAL (Figure 1). After removing the duplicate articles, we evaluated the abstracts of 303 articles. Of these articles, 292 were excluded because they did not meet the inclusion criteria of this systematic review. Subsequently, we read the full text of each of the remaining 11 articles. Five articles were then excluded because the RCT did not include patients with AKI (*n* = 3) or because it was not an RCT (*n* = 2). Thus, 6 RCTs involving 1,292 participants were used in the meta-analysis.^(6,7,11-14)^

**Figure 2:**
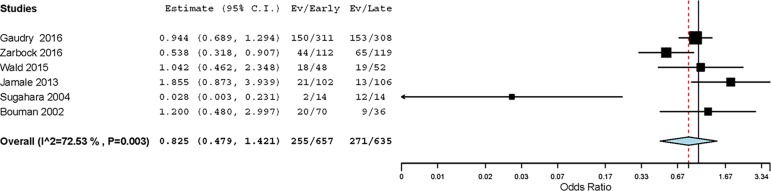
Forest plot showing the effect of early renal replacement therapy initiation on mortality at the longest follow-up in patients with acute kidney injury.

Table 1 summarizes the characteristics of the RCTs included. Notably, 2.3% of the patients in the early initiation group and 32.6% of the patients in the delayed initiation group never received RRT (Table 1). The criteria for early or delayed RRT initiation, RRT dose, the modality used, the cumulative urine output, the fluid balance 24 hours before RRT starting and the baseline creatinine varied among the studies (Table 1S - supplementary material). The quality of the RCTs is shown in Figure 1S and 2S (supplementary material). Three RCTs were considered to have a lower risk of bias because they showed a low risk of bias in all of the domains assessed,^(6,7,13)^ excluding the blinding of personnel, patients, or outcome assessors. In fact, only one RCT used blinding of the outcome assessment.^(13)^ In three RCTs,^(11,12,14)^ the generation of the randomization list and the allocation concealment were adequate; however, in the other RCTs, insufficient information on the randomization method or allocation concealment was reported.^(11,12,14)^ One RCT was considered at a high risk for other biases because the sample size was not calculated *a priori,*^(12)^ and another RCT was considered at an unclear risk of bias because patients with an indication for immediate RRT by either the intensivist or the nephrologist were excluded, regardless of the predefined indications of RRT.^(14)^

**Table 1:** Characteristics of the studies included

Study	Design	Population	Patients (N)			Criteria for initiation of RRT		Modality	Never received RRT	
			Total	Early	Late	Early	Late		Early N (%)	Late N (%)
Zarbock et al.^(6)^	RCT, single-center	AKI in mixed patients	231	112	119	Within 8 hours after diagnosis of stage 2 AKI by KDIGO	Within 12 hours after diagnosis of stage 3 AKI by KDIGO	Continuous	0 (0)	11 (9.2)
Gaudry et al.^(7)^	RCT, multicenter	AKI in mixed patients	619	308	311	Within 6 hours after diagnosis of stage 3 AKI KDIGO	Oliguria or anuria > 72 hours or urea > 112mg/dL or K > 6mmol/L or pH < 7.15 or pulmonary edema	IHD or Continuous	6 (1.9)	154 (49.5)
Bouman et al.^(11)^	RCT, multicenter	AKI in mixed patients	106	70	36	Within 12 hours after randomization*	Urea > 40mmol/L or K > 6.5mmol/L or pulmonary edema	Continuous	0 (0)	6 (17)
Sugahara et al.^(12)^	RCT, single-center	AKI after cardiac surgery	28	14	14	Urine output < 30mL/h for three hours	Urine output < 20mL/hour for two hours	Continuous	0 (0)	0 (0)
Jamale et al.^(13)^	RCT, single-center	AKI in mixed patients	208	102	106	Urea > 70mg/dL or creatinine > 7mg/dL	Clinically indicated by the nephrologist	IHD	9 (8.8)	18 (17)
Wald et al.^(14)^	RCT, multicenter	AKI in mixed patients	100	48	52	Within 12 hours after randomization**	K > 6mmol/L or HCO_3_ < 10mmol/L or PaO_2_/FiO_2_ < 200 and pulmonary edema	IHD or Continuous	0 (0)	19 (36.5)

RRT - renal replacement therapy; RCT - randomized controlled trial; AKI - acute kidney injury; K - potassium; IHD - intermittent hemodialysis; HCO_3_ - bicarbonate; PaO_2_/FiO_2_ - fraction of inspired oxygen/arterial oxygen pressure; KDIGO - Kidney Disease: Improving Global Outcomes.

* urine output < 30mL/h for > 6 hours + creatinine clearance < 20mL/min + mechanical ventilation;

** kidney dysfunction (defined as a serum creatinine ≥ 100µmol/L for women or ≥ 130µmol/L for men) + severe AKI + absence of urgent indications + low likelihood of volume-responsive AKI.

### Primary endpoint

**Figure 3:**
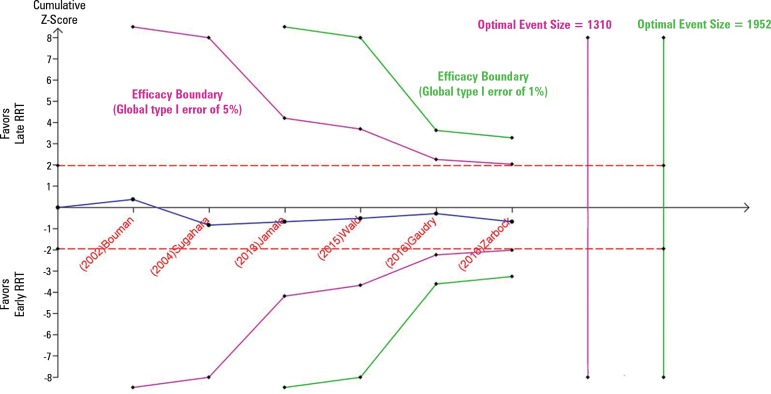
Trial sequential analysis assessing the effect of early renal replacement therapy initiation on mortality at the longest follow-up. The cumulative meta-analysis with 526 in-hospital deaths (blue line) did not cross the efficacy monitoring boundary for the primary outcome (i.e., the overall type I error is > 5% [purple line]). Considering a global type I error of 1%, the cumulative meta-analysis also did not cross the efficacy monitoring boundary, and the optimal event size of 1952 (green line) was not reached. The optimal event size is the event size needed for a very precise meta-analysis (which is at least as large as that for a single optimally powered randomized controlled trial). RRT - renal replacement therapy.

All RCTs were considered for the analysis of the primary endpoint. Two hundred and fifty-five out of 657 (38.8%) patients assigned to early RRT initiation and 271 out of 635 (42.6%) assigned to delayed RRT initiation died during the longest follow-up reported (OR 0.82; 95%CI, 0.48 - 1.42; p = 0.488) (Figure 2). There was moderate-to-high heterogeneity, (*I^2^* = 72%; p = 0.003) explained by the following two RCTs: one single-center RCT with a large effect size^(6)^ and another RCT conducted in a cohort of surgical ICU patients.^(12)^ The funnel plot was visually asymmetric, suggesting that publication bias may have affected the results (Figure 3S).

**Figure 4:**
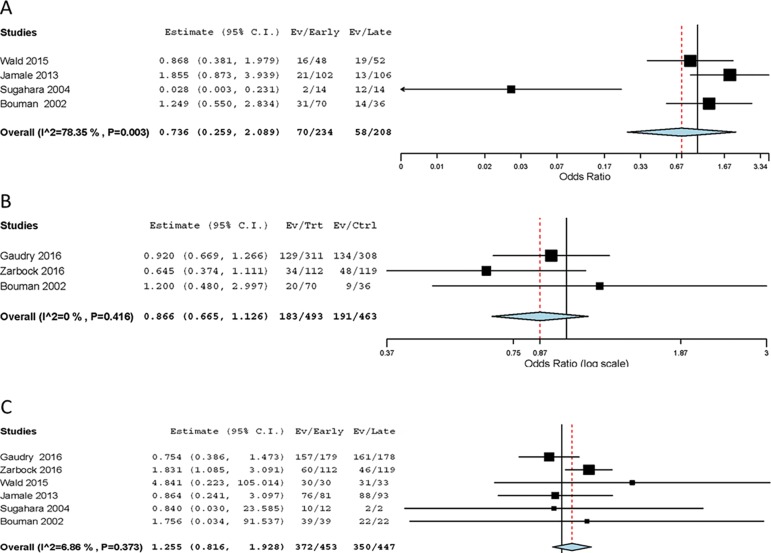
Forest plot showing the effect of early renal replacement therapy initiation on (A) in-hospital mortality; (B) 28-day mortality; and (C) renal function recovery at the longest follow-up in patients with acute kidney injury.

According to the meta-regression, only the percentage of patients receiving a continuous form of RRT in the early arm was significantly associated with mortality at the longest follow-up (p = 0.012) (Figure 4S). The total number of deaths was 526, which is lower than the optimal event size (1310 events); that is, the TSA indicated an overall type I error of greater than 5% for the meta-analysis result. Additionally, when a more conservative type I error of 1% is used, the number of events is still insufficient, and the cumulative meta-analysis did not cross the efficacy monitoring boundary (Figure 3). We classified the quality of evidence generated by the meta-analysis for the primary outcome as low due to the risk of bias of the included studies and the inconsistency.

### Secondary endpoints

There was no difference in the in-hospital mortality (Figure 4A). The high heterogeneity was explained by one RCT conducted in a cohort of surgical ICU patients.^(12)^ Additionally, there was no difference in the 28 day mortality and renal function recovery at the longest follow-up (Figure 4B and 4C). According to the meta-regression, the time between randomization and RRT initiation in the early initiation arm (p = 0.030) and the percentage of patients receiving a continuous method of RRT in the early initiation arm (p = 0.046) was significantly associated with renal function recovery at the longest follow-up (Figure 5S).

Regarding complications potentially related to the AKI or RRT, there was no difference in bleeding or thrombosis (Figure 6S). Nevertheless, there was an increased risk of catheter-related bloodstream infection in patients in the early initiation arm (OR 1.77; 95%CI, 1.01 - 3.11; p = 0.047) (Figure 6S).

### Subgroup analyses

There was no difference in mortality at the longest follow-up when assessing RCTs using either continuous RRT or any type of RRT (the p value for subgroup differences = 0.140) (Figure 7SA) or RCTs with a lower risk of bias or higher risk of bias (the p value for subgroup differences = 0.410) (Figure 7SB).

In studies using only continuous forms of RRT, there was an increased rate of renal function recovery at the longest follow-up in the early initiation group (p = 0.025) (Figure 8SA). There was no relationship between the time of RRT initiation and renal function recovery at the longest follow-up when considering studies using any type of RRT (the p value for subgroup differences = 0.05) (Figure 8SA). A pooled analysis of the trials with a lower risk of bias showed similar effects on renal function recovery at the longest follow-up than the pooled analysis of trials with a higher risk of bias (the p value for subgroup differences = 0.60) (Figure 8SB).

## DISCUSSION

This updated systematic review and meta-analysis of RCTs of RRT in ICU patients with AKI found that early RRT initiation is not superior to delayed RRT initiation with regard to any mortality outcome. Catheter-related bloodstream infections occurred more often with early RRT initiation, but there were no differences in bleeding or thrombotic events.

Our review has a number of strengths. First, our search strategy was comprehensive, including electronic databases, clinical trial registries, and hand-searching the reference lists of the included studies and other relevant studies. Second, we conducted eligibility assessment and data extraction in duplicate. Third, we used several exploratory analyses using meta-regressions. Fourth, we evaluated the reliability and conclusiveness of the available evidence with a formal TSA method. Finally, we evaluated the quality of evidence using the GRADE system.

The current concern over the best time to initiate RRT in AKI patients represents an important gap in the literature and contributes to wider variations in clinical practice regarding when to start dialysis in critically ill patients.^(15,16)^ The lack of consensus regarding the definition of 'early' in RRT makes it even more difficult, and at times, hampers the interpretation of findings. In the majority of RCTs evaluating the timing of RRT in patients with AKI, the patients were randomized from a point in time prompted by the development of AKI as diagnosed by the Acute Kidney Injury Network (AKIN), the KDIGO or the Risk, Injury, Failure, Loss of kidney function and End-stage kidney disease (RIFLE) criteria. However, these criteria were never intended to determine the need for RRT.^(14,17,18)^

Additionally, another important aspect to consider is the vast heterogeneity of patients and RRT procedures among the RCTs, which may vary from sepsis, postoperative, leptospirosis, mixed populations and continuous renal replacement therapies (CRRT) or intermittent hemodialysis (IHD), with different doses and ultrafiltration rates, making the interpretation of results even more challenging.^(19-23)^ Recently, two new RCTs evaluating the RRT timing in AKI patients were published, with important differences in the results.^(6,7)^ While the 'Early *versus* LAte Initiation of renal replacement therapy in critically ill patients with acute kidNey injury' (ELAIN) trial randomized a majority of surgical patients undergoing CRRT to early versus delayed RRT initiation,^(6)^ the 'Artificial Kidney Initiation in Kidney Injury' (AKIKI) trial randomized a mixed population with a large percentage of patients with sepsis and used IHD in the majority of the cases,^(7)^ making it difficult to compare the results between these two investigations. Additionally, the early initiation RRT criteria in the AKIKI trial (within 6 hours after fulfilling AKIN stage 3 criteria) were relatively late and almost similar to the late initiation RRT criteria in the ELAIN trial (within 12 hours after fulfilling AKIN stage 3 criteria).

The rationale for early RRT initiation is based both on physiological reasoning and clinical data. Earlier RRT initiation, theoretically, could improve the management of uremia, acidemia, electrolyte imbalances and extracellular volume accumulation. Studies observed that an early approach might be associated with improved survival in patients with refractory septic shock, possibly due to the pro-inflammatory effects caused by uremic solutes.^(24-26)^ Additionally, observational studies demonstrate an association between mortality, fluid accumulation and increased urea levels at the time of RRT initiation.^(27,28)^ However, demonstrating the benefits of early RRT initiation in prospective clinical trials may be difficult due to the heterogeneity of the criteria used to define both the AKI stages and defining early and late initiation.

An important consideration for clinicians is whether the triggers used for initiating RRT in the meta-analyzed RCTs are in fact translatable to routine bedside practice.^(29)^ In fact, in all but one RCT, there were patients in the delayed ignition arm who never received RRT based on the pre-established triggers. Indeed, one may infer that some patients in the early initiation arm may have received RRT unnecessarily and would have recovered spontaneously.^(29)^ With the absence of objective markers to inform the need for RRT, early RRT initiation will inevitably enroll patients who might never require RRT. Based on the data provided in the present study, this approach could result in a small increase in the risk of catheter-related bloodstream infections.

The results of this meta-analysis should be interpreted within the context of the included RCTs. Systematic reviews are subject to the overall quality of the studies, and publication bias can occur. Additionally, there was a large variation in the trials regarding the moment of RRT initiation, the diagnosis of AKI, the duration of follow-up and the type of RRT. The fact that practically all secondary outcomes were only reported by some eligible trials is another limitation. Indeed, unreported outcomes could lead to the overestimation of effects in the meta-analyses. Additionally, the presence of moderate-to-high heterogeneity in some analyses decreases the strength of the findings. Finally, we classified the quality of evidence generated by this meta-analysis as low. The main reasons for downgrading the quality of evidence include the risk of bias of the included studies and the inconsistency. We considered the results inconsistent because of the presence of a moderate to high heterogeneity. Furthermore, despite our comprehensive search, we cannot completely rule out publication bias because the funnel plot was visually asymmetric, although interpretation of the funnel plot was hampered due to the low number of RCTs. Moreover, the cumulative meta-analysis did not achieve the optimal event size with a global type I error rate of 5% or 1%. Thus, there is still some chance that future research may contradict the current findings. Finally, a recent meta-analysis including the same six studies was published.^(30)^ Compared to this meta-analysis, the present study assessed a greater number of endpoints and utilized different exploratory analyses including the use of meta-regressions to assess the impact of the various covariates on the outcome and the TSA report, thus assessing the reliability and conclusiveness of the available evidence.

## CONCLUSION

This systematic review and meta-analysis suggests that early initiation of renal replacement therapy does not improve the survival of intensive care unit patients with acute kidney injury. However, the quality of the current evidence is low and insufficient for determining definitive and reliable conclusions. Additionally, the criteria used for defining early and delayed initiation of renal replacement therapy were heterogeneous among the studies, which can impact the accuracy of our findings.

### Authors' contributions

FT Moreira and A Serpa Neto conceived the idea for this study. A Serpa Neto designed the search strategy and performed the data analyses. FT Moreira and RCF Chaves selected the eligible studies to be included in this review. FT Moreira and A Serpa Neto wrote the manuscript, with contributions from H Palomba, MJ Schultz and C Bouman. All of the authors have read and approved the final manuscript.
