# Supplementary material for: Early versus delayed initiation of renal replacement therapy for acute kidney injury: an updated systematic review, meta-analysis, meta-regression and trial sequential analysis of randomized controlled trials
Source: Rev Bras Ter Intensiva. 2018;30(3):376–84. doi: 10.5935/0103-507X.20180054 (PMC6180467; doi:10.5935/0103-507X.20180054)
Supplement: Supplementary file 1 [file rbti-30-03-0376-suppl1.pdf]

Early *versus* delayed initiation of renal replacement therapy for acute kidney injury: an updated systematic review, meta-analysis, meta-regression and trial sequential analysis of randomized controlled trials

Início precoce em comparação ao início tardio da terapia de substituição renal para lesão renal aguda: revisão sistemática atualizada, metanálise, metarregressão e análise sequencial de ensaios clínicos randomizados e controlados

Fabio Tanzillo Moreira[1], Henrique Palomba[1], Renato Carneiro de Freitas Chaves[1], Catherine Bouman[2], Marcus Josephus Schultz[2,3], Ary Serpa Neto[1,2]

Table 1S - Characteristics of the included studies and patients at the beginning of renal replacement therapy

Study	Type of RRT		Fluid balance (mL)		Dose of RRT (mL/kg/h)		Serum creatinine (mg/dL)		Urine output (mL)	
	Early	Late	Early	Late	Early	Late	Early	Late	Early	Late
Zarbock et al.[6]	CVVHDF	CVVHDF	6811 (3897 - 10,189)	6334 (3951 - 10,700)	26.6 ± 4.7	26.6 ± 5.8	1.9 ± 0.6	2.4 ± 1.0	445 (175 - 807)	270 (112 - 670)
Gaudry et al.[7]	IHD, CRRT	IHD, CRRT	NR	NR	NR	NR	3.3 ± 1.4	5.3 ± 2.3	NR	150 (50 - 600)
Bouman et al.[11]	CVVHF	CVVHF	NR	NR	52 ± 16 (high) 20 ± 4 (low)	19 ± 4	NR	NR	NR	NR
Sugahara et al.[12]	CVVHD	CVVHD	NR	NR	NR	NR	2.9 ± 0.2	3.0 ± 0.2	696 ± 24	432 ± 24
Jamale et al.[13]	IHD	IHD	NR	NR	NR	NR	7.4 ± 5.3	10.4 ± 3.3	429 ± 388	376 ± 350
Wald et al.[14]	IHD, SLED, CRRT	IHD, SLED, CRRT	5144 (2440 - 7006)	4821 (3149 - 7761)	28.6 ± 8.0	24.7 ± 28.6	3.7 ± 1.3	4.6 ± 2.2	400 (211 - 568)	265 (80 - 755)

RRT - renal replacement therapy; CVVHF - continuous venovenous hemofiltration; NR - not reported; CVVHD - continuous venovenous hemodialysis; IHD - intermittent hemodialysis; SLED - sustained low efficiency dialysis; CRRT - continuous renal replacement therapy; CVVHDF - continuous venovenous hemodiafiltration.

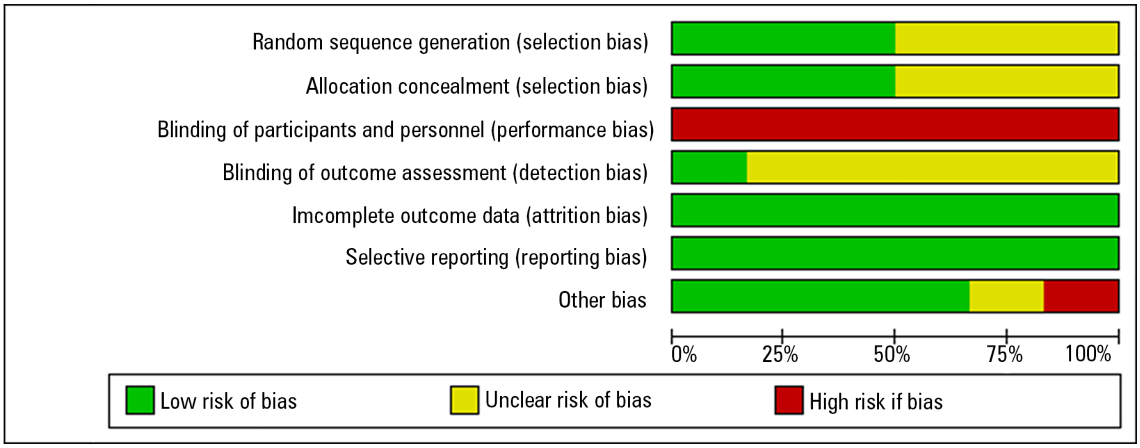

Figure 1S - Risk of bias graph.

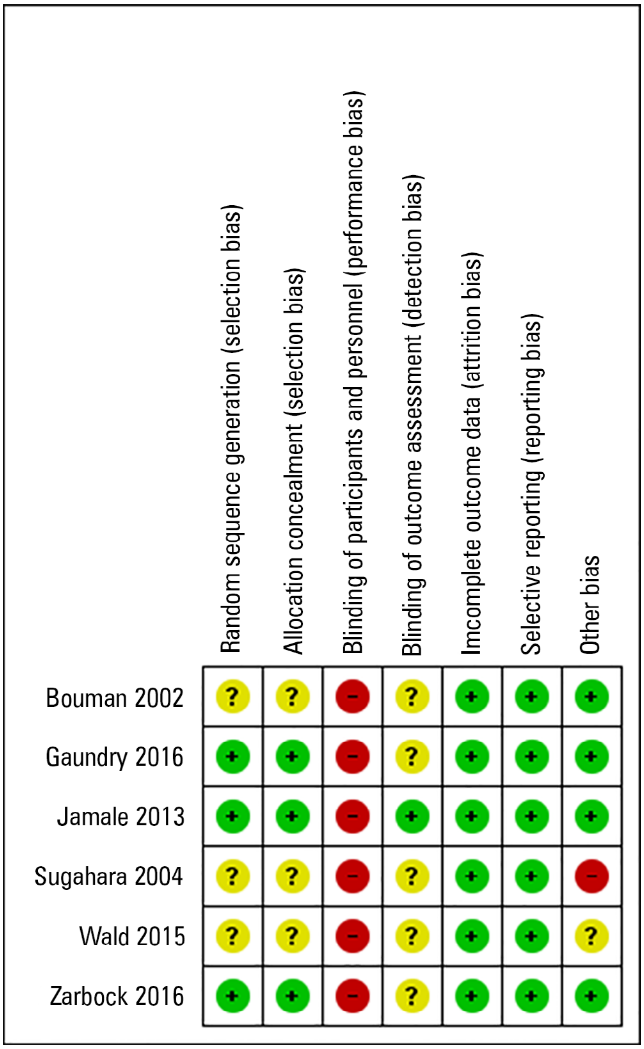

Figure 2S - Risk of bias summary.

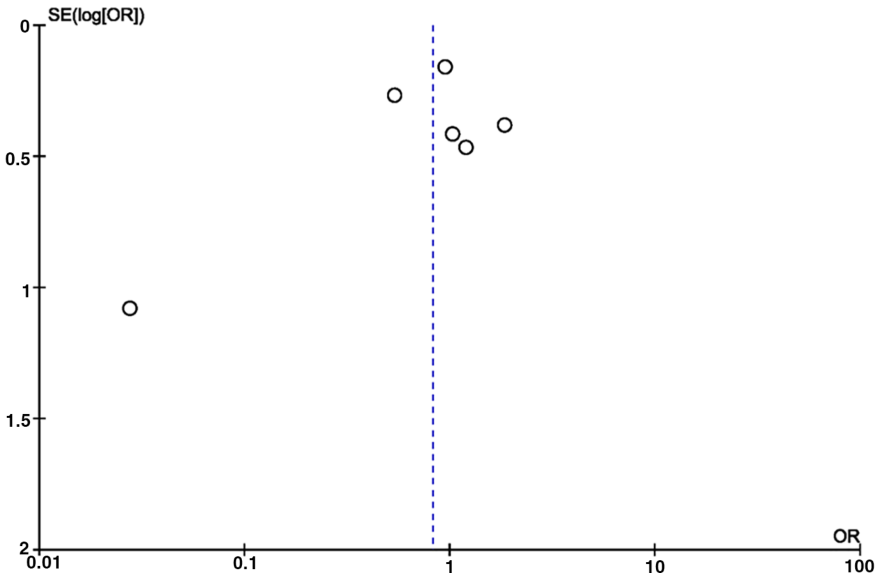

Figure 3S - Funnel plot for the primary outcome.

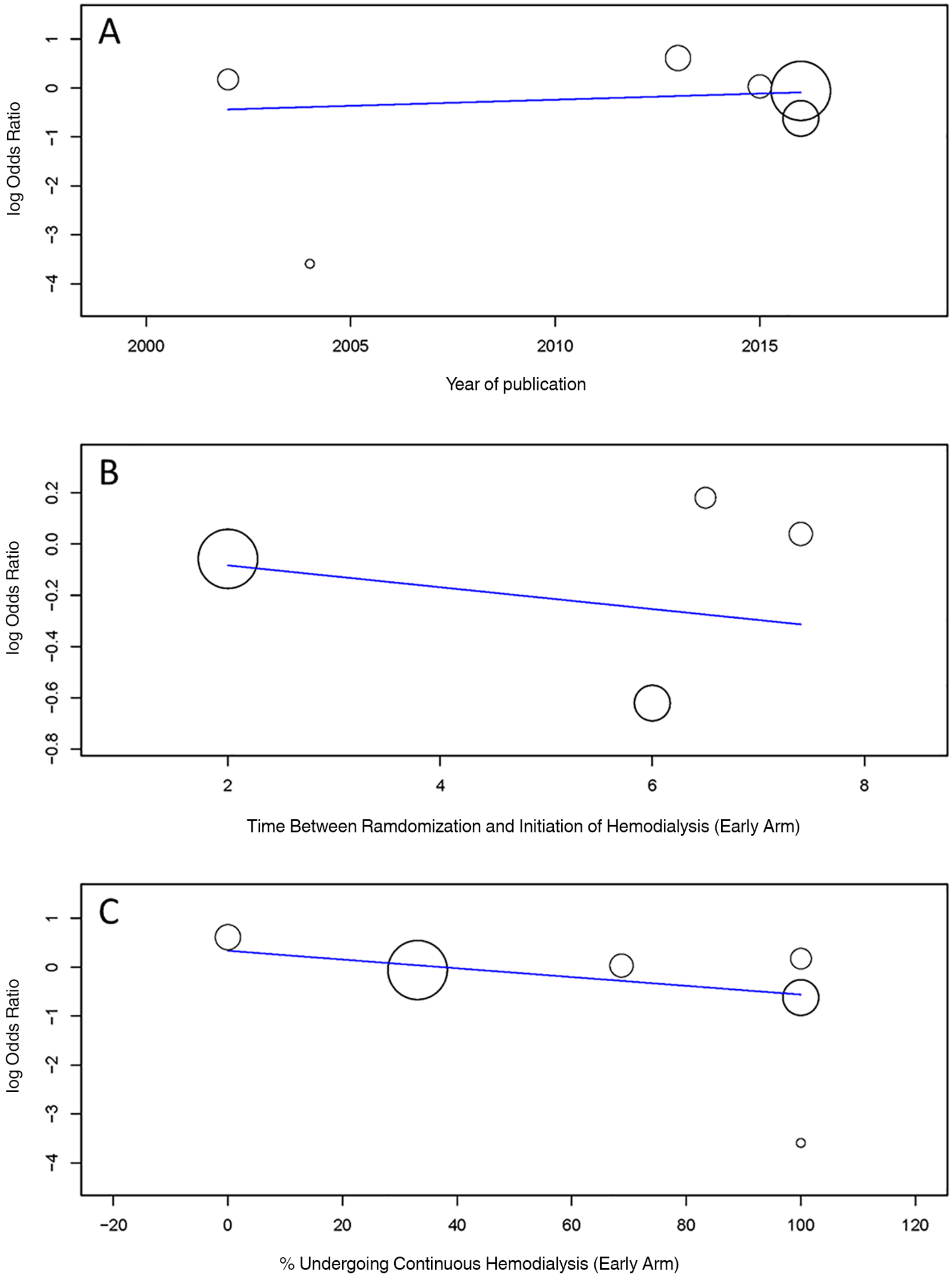

Figure 4S - Meta-regression analyses for mortality at the longest follow-up using (A) the publication year, (B) the time between randomization and initiation of renal replacement therapy in the early arm, and (C) the percentage of patients receiving the continuous method of renal replacement therapy in the early arm as covariates.

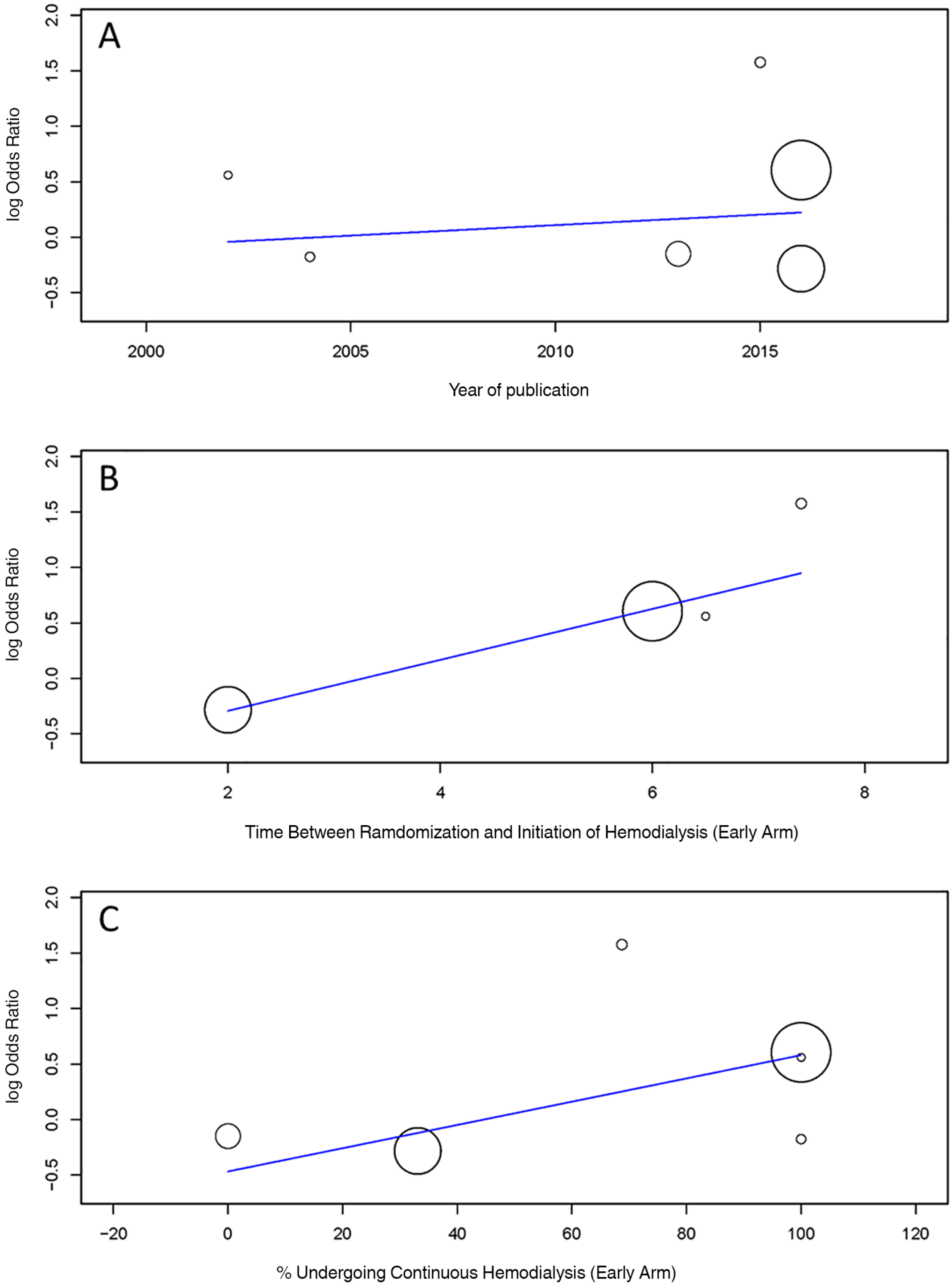

Figure 5S - Meta-regression analyses for renal function recovery at the longest follow-up using (A) the publication year, (B) the time between randomization and initiation of renal replacement therapy in the early arm, and (C) the percentage of patients receiving the continuous method of renal replacement therapy in the early arm as covariates.

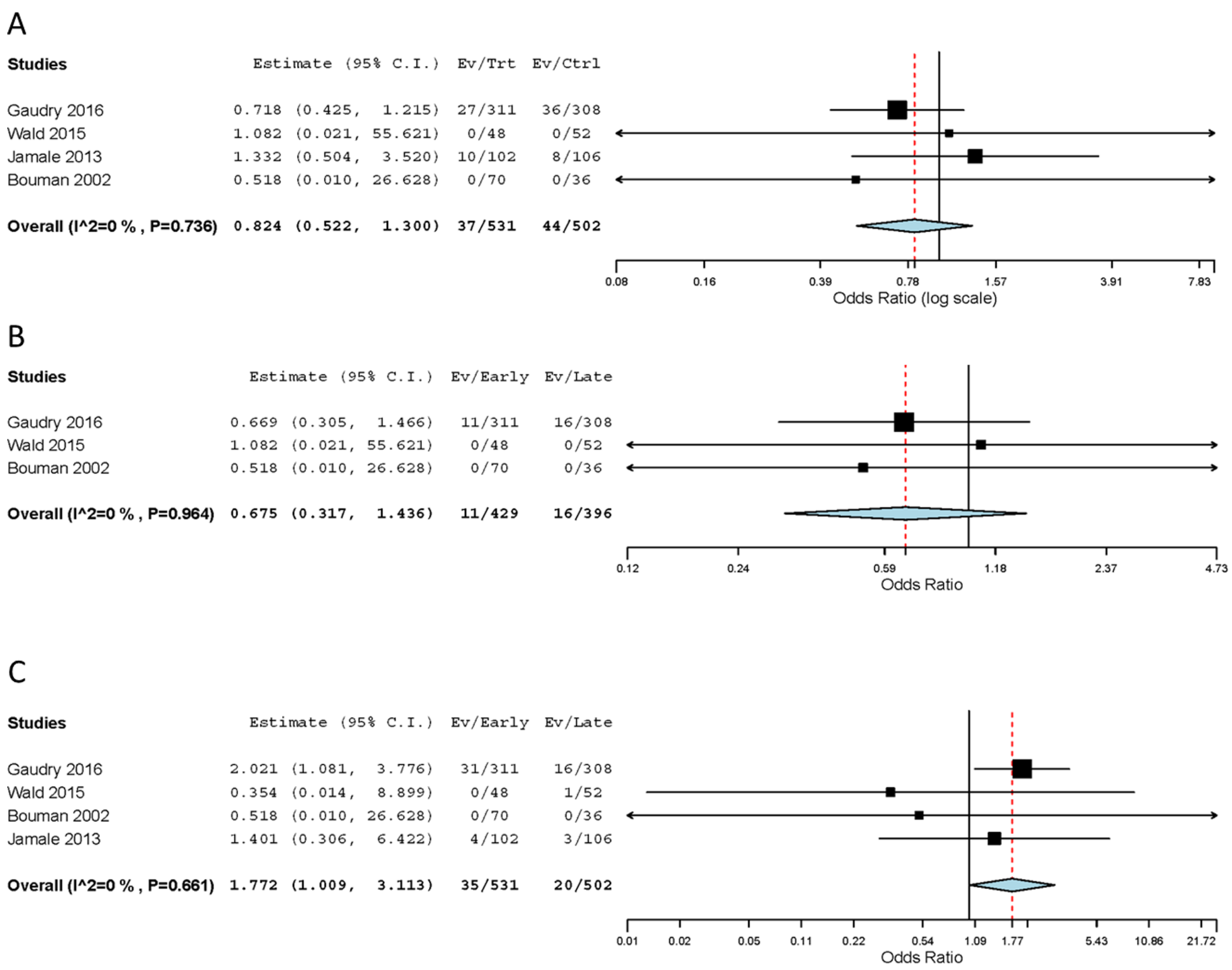

Figure 6S - Forest plots for (A) bleeding, (B) thrombosis, and (C) catheter-related bloodstream infections.

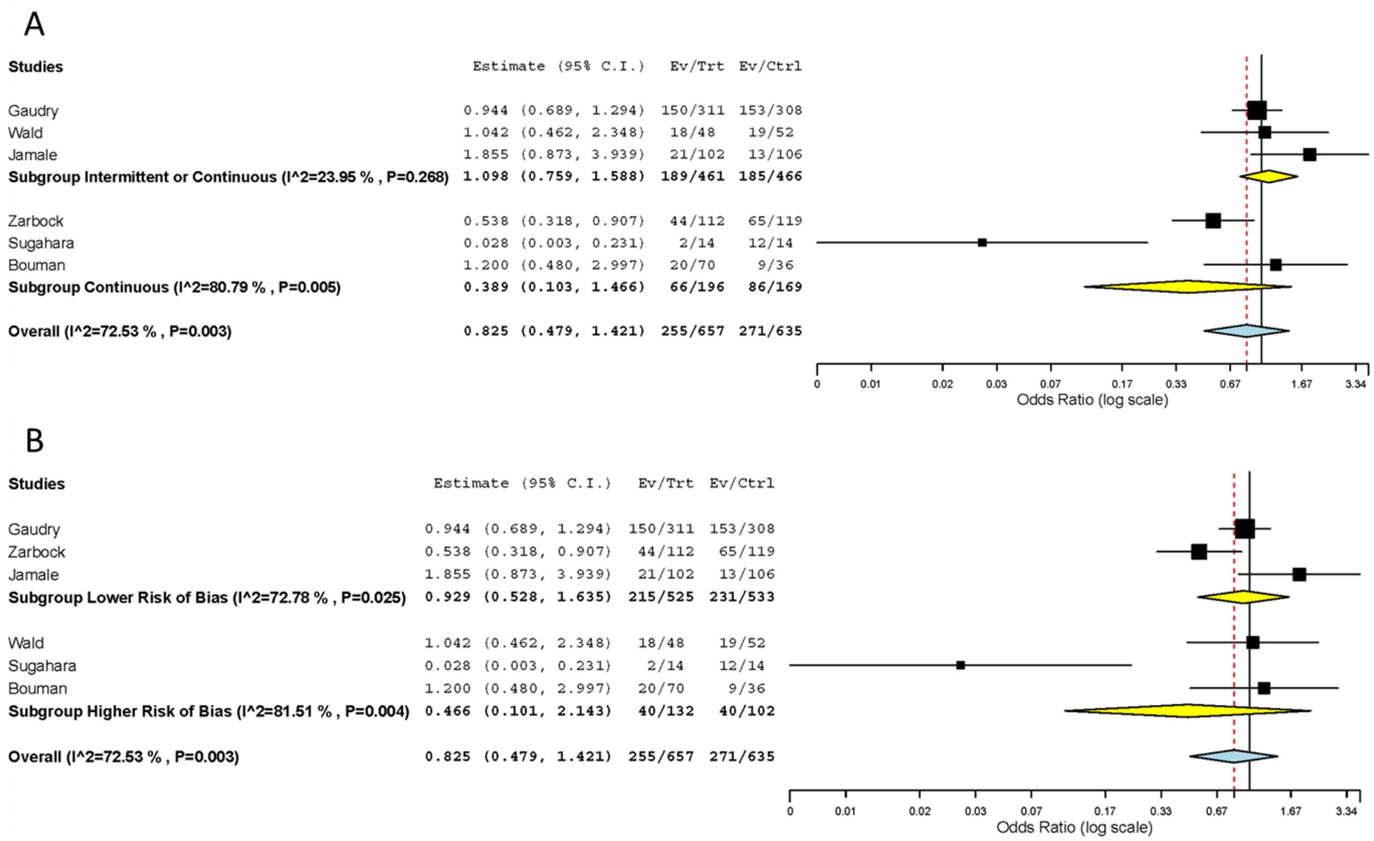

Figure 7S - Forest plots showing the effects of early renal replacement therapy initiation on mortality at the longest follow-up according to the (A) type of renal replacement therapy used (exclusively continuous *vs* intermittent or continuous) and to the (B) risk of bias (lower *vs* higher).

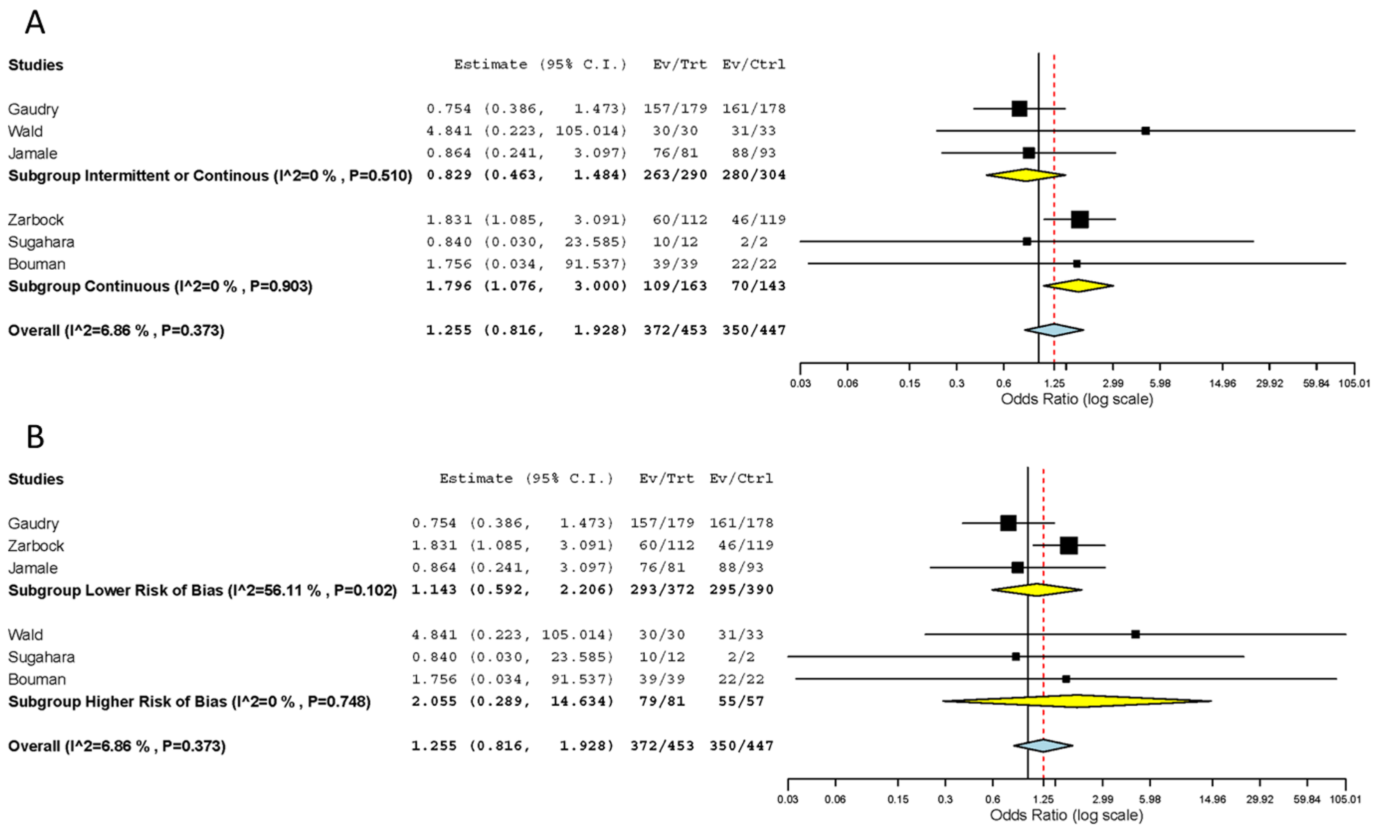

Figure 8S - Forest plots showing the effects of early renal replacement therapy initiation on renal function recovery at the longest follow-up according to the (A) type of renal replacement therapy used (exclusively continuous *vs* intermittent or continuous) and to the (B) risk of bias (lower *vs* higher).
